# Broadband biphoton generation and statistics of quantum light in the UV-visible range in an AlGaN microring resonator

**DOI:** 10.1038/s41598-017-11617-y

**Published:** 2017-09-12

**Authors:** Francesco De Leonardis, Richard A. Soref, Mohammad Soltani, Vittorio M. N. Passaro

**Affiliations:** 1 Dipartimento di Ingegneria Elettrica e dell’Informazione, Politecnico di Bari Via Edoardo Orabona n. 4, 70125 Bari, Italy; 2 Department of Engineering, The University of Massachusetts, Boston, Massachusetts 02125 USA; 3 Raytheon BBN Technologies, 10 Moulton Street, Cambridge, MA 02138 USA

## Abstract

We present a physical investigation on the generation of correlated photon pairs that are broadly spaced in the ultraviolet (UV) and visible spectrum on a AlGaN/AlN integrated photonic platform which is optically transparent at these wavelengths. Using spontaneous four wave mixing (SFWM) in an AlGaN microring resonator, we show design techniques to satisfy the phase matching condition between the optical pump, the signal, and idler photon pairs, a condition which is essential and is a key hurdle when operating at short wavelength due to the strong normal dispersion of the material. Such UV-visible photon pairs are quite beneficial for interaction with qubit ions that are mostly in this wavelength range, and will enable heralding the photon-ion interaction. As a target application example, we present the systematic AlGaN microresonator design for generating signal and idler photon pairs using a blue wavelength pump, while the signal appears at the transition of ytterbium ion (^171^Yb^+^, 369.5 nm) and the idler appears in the far blue or green range. The photon pairs have minimal crosstalk to the pump power due to their broad spacing in spectral wavelength, thereby relaxing the design of on-chip integrated filters for separating pump, signal and idler.

## Introduction

Entangled photon generation is an essential ingredient in quantum information processing. Traditional sources produce such photon pairs using spontaneous parametric down conversion (SPDC) in χ^(2)^ nonlinear materials^1, 2^, and more recently, spontaneous four-wave mixing (SFWM) in χ^(3)^ standard optical fibers^3–5^ and photonic crystal fibers^6, 7^. However, the common drawback for all these sources is their lack of scalability to chip-scale platforms that are needed to handle a large number of qubits. Recently, integrated silicon photonics and microring resonators in this material platform have proven their potential for the generation of correlated photon pairs through the SFWM effect at telecommunication wavelengths promising scalable long distance quantum key distribution (QKD)^8–20^.

While the wavelength host for quantum communication is the telecom band which justifies the use of silicon photonics, quantum computing platforms based on ion-traps are functional in the ultraviolet and visible (UV-vis) range^21–25^, mainly due to the ion’s transition wavelengths that are mostly in this wavelength spectrum. Therefore, a photonic integrated circuit (PIC) platform capable of generating correlated pairs at these wavelengths to interact with ions is needed. A PIC platform for the UV-vis wavelength range requires wide bandgap materials to be optically and preferably crystalline to avoid excess optical absorption, background broadband Raman noise, and autofluorescence^26^.

In this context, an integrated photonic platform made of crystalline AlGaN^26^ with its wide bandgap and optical transparency at short wavelengths as well as its large 3^rd^ order nonlinearity is promising to enable compact microring resonators with strong nonlinear interaction. However, a challenge in SFWM is to satisfy the phase matching between the optical pump, signal, and idler wavelengths. This challenge is even more difficult at visible and UV wavelengths where the optical material shows strong normal dispersion. Another common problem in SFWM is to isolate the signal/idler photons from the strong pump photons. This requires making integrated photonic filters with strong rejection bands to suppress the pump, or to make the signal/idler wavelengths quite far from the pump wavelength such that the filter design becomes easier.

In this paper, we show techniques for efficient SFWM generation of broadly spaced correlated photon pairs in the UV-vis range in an AlGaN microring resonator by satisfying the phase matching condition between the pump, the signal, and the idler. We show that by using the higher-order modes of the resonator, we can overcome the strong normal dispersion of the resonator material at the UV-visible, and provide the energy and momentum matching conditions for the generation of photon pairs at the UV-visible range. We discuss the spectral properties of the pairs and their generation rates under different conditions of the pump and the resonator parameters.

The paper is organized as follows. A theoretical formulation is reported in the Theory Section to estimate the correlation functions among signal and idler photons. In particular, the aim of this section is to describe the main physical effects that can influence the biphoton flux, the cross-correlation function and the generated frequencies. Design guidelines for the ring resonator are derived in the Results Section, and parametric simulations are performed to theoretically demonstrate the generation of entangled photons in the UV-vis wavelength range. The Conclusion Section summarizes with concluding remarks.

## Theory

**Figure 1:**
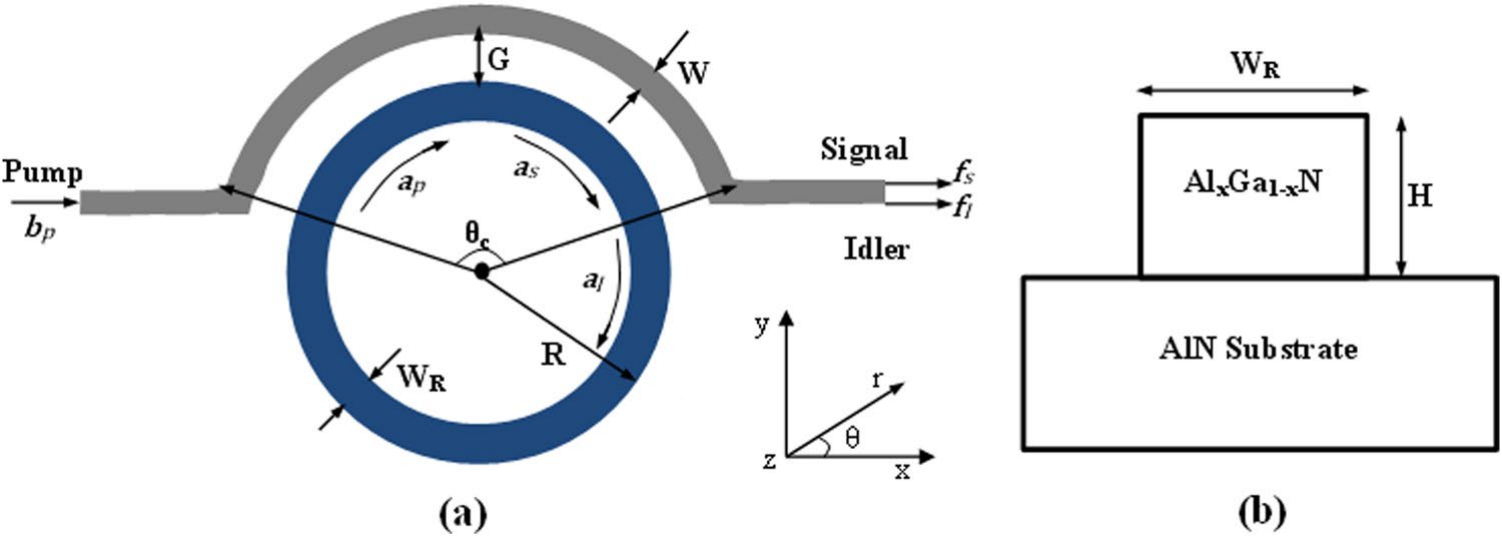
(**a**) Schematic architecture of entangled photon source based on an Al_x_Ga_1−x_N microring resonator with pulley coupler; (**b**) The cross section of the Al_x_Ga_1−x_N ring resonator on an AlN substrate platform.

Figure 1(a) shows the structure of an AlGaN ring resonator on an AlN substrate and coupled to an external waveguide. The input optical pump with power *P*_*in*_ in the bus waveguide is coupled to microring resonator by means of a pulley directional coupler characterized by a gap, *G*, and a coupler angle *θ*_*c*_. The pulley coupling provide more degree of freedom when a stronger waveguide-resonator interaction is needed^27, 28^. Then the pump wave (*p*) launched into the resonator produces a pair of signal (*s*) and idler (*I*) photons which are trasmitted out into the coupling waveguide. The plot indicates the fundamental field operator involved in the process.

The Al_x_Ga_1−x_N resonator consists of a fully-etched waveguide cross-section with width, *W*_*R*_, and height, *H*, sitting on AlN substrate, and with a top cladding of air as sketched in Fig. 1(b). The cross section of the outer bus waveguide has the same height, but different width (*W*).

The interaction among the three cavity modes at frequencies *ω*_*o*,*i*_ (*i* = *p*, *s*, *I*) can be described by the Hamiltonian *H* = *H*_0_ + *H*_*in*_, where *H*_0_ describes the passive cavity modes coupled to the bus waveguide:

1 $${H}_{0}={\sum }_{i=p,s,I}\{\hslash {\omega }_{0,i}{a}_{i}^{\dagger }{a}_{i}+\hslash \sqrt{{\Gamma }_{c,i}}[{a}_{i}^{\dagger }{b}_{i}+{b}_{i}^{\dagger }{a}_{i}]+\hslash \sqrt{{\Gamma }_{l,i}}[{a}_{i}^{\dagger }{u}_{i}+{u}_{i}^{\dagger }{a}_{i}]\}$$

2where ${a}_{i}^{\dagger }$ and *a*_*i*_ (*i* = *p*, *s*, *I*) are the intracavity photon annihilation and creation operators, respectively, and normalized such that ${a}_{i}^{\dagger }{a}_{i}$ represents the photon number operator. Similarly, the annihilation and creation photon operators in the bus waveguide are ${b}_{i}^{\dagger }$ and *b*_*i*_, respectively, wherein ${b}_{p}^{\dagger }{b}_{p}$ represents the operator of the input pump photon flux, and is $\langle {b}_{s}^{\dagger }{b}_{s}\rangle =\langle {b}_{I}^{\dagger }{b}_{I}\rangle =0$, since only the pump is launched into the cavity. The noise operators ${u}_{i}^{\dagger }$, and *u*_*i*_ are associated with the cavity intrinsic loss and the interaction of the cavity with the background thermal photons \reservir^29^. Thus, the coefficients *Γ*_*c*,*i*_, and *Γ*_*l*,*i*_ represent the external coupling rate and the loss rate of the cavity mode at *ω*_*o*,*i*_ (*i* = *p*, *s*, *I*), respectively. In particular, the decay rate due to losses can also be estimated as a function of the unloaded linear loss quality factor (*Q*_*l*,*i*_) by means of the relationship *Γ*_*l*,*i*_ = ω_*o*,*i*_/*Q*_*l*,*i*_. In addition, the coupling rate coefficient depends on the coupling factor ${\kappa }_{i}^{2}$ by means of ${{\Gamma }}_{c,i}=({\kappa }_{i}^{2}{v}_{g,i}/{L}_{cavity})$, where *L*_*cavity*_ is the physical circumference cavity length_,_ and *ν*_*g*,*i*_ represents the group velocity of the *i*-th wave involved in the process.

In Eq. (2), the first term on the right side describes the self-phase modulation of the pump mode, while the second is responsible for the cross-phase modulation (XPM) between the pump and the signal and the idler modes, and the last term in Eq. (2) governs the SFWM induced by Kerr nonlinearity. The Kerr-induced coupling strength coefficient in the last term of *H*_*in*_ can be estimated as^30^:

3 $$\zeta =\frac{{n}_{2}{c}_{0}\hslash {\omega }_{p}\sqrt{{\omega }_{s}{\omega }_{I}}}{{n}_{s}{n}_{I}}\frac{{\iint }_{core}{e}_{p}(r,y){e}_{p}(r,y){e}_{s}^{\ast }(r,y){e}_{I}^{\ast }(r,y)drdy}{{L}_{cavity}{\prod }_{i=p,p,s,I}\sqrt{\iint {|{e}_{i}(r,y)|}^{2}drdy}}\delta (2{m}_{p}-\,{m}_{I}-{m}_{s})$$

where *n*_2_ and *c*_0_ are the Kerr coefficient at the pump frequency and the vacuum light velocity, respectively. The terms *n*_*i*_ represents the Al_x_Ga_1−x_N refractive index at the wavelength of the *i*-th mode (*i* = *p*, *s*, *I*) involved in the process. The term *m*_*i*_ is the angular momentum of the *i*-th optical mode propagating inside the ring resonator. The electric field of the optical modes in the cylindrically symmetric microring can be expressed as ${e}_{i}({\boldsymbol{r}})={e}_{i}(r,y){e}^{j{m}_{i}\theta }$. The coefficients *η*_*ij*_ can be approximated by means of the relationship *η*_*p*_ ≅ *η*_*ps*_ ≅ *η*_*pI*_ ≅ *η* ≅ *ζ*. The Kronecker’s delta function *δ*(2*m*_*p*_ − *m*_*I*_ − *m*_*s*_) is produced by the integration of the electric field distribution over the cylindrical coordinate *θ*, and indicates that the coupling strength coefficient is non-zero only when 2*m*_*p*_ = *m*_*I*_ + *m*_*s*_ (momentum-conservation condition).

Using the slowly-varying operator, ${\bar{a}}_{i}(t)={a}_{i}(t){e}^{j{\omega }_{0,i}t},\,$ (*i* = *p*, *s*, *I*) the Heisenberg motion equation becomes:

4 $$\frac{d{\bar{a}}_{p}}{dt}=-{\bar{\Gamma }}_{p}{\bar{a}}_{p}-j\eta {\bar{a}}_{p}^{\dagger }{\bar{a}}_{p}^{2}-j2\zeta {\bar{a}}_{p}^{\dagger }{\bar{a}}_{s}{\bar{a}}_{I}-j{\gamma }_{p}{\bar{b}}_{p}-j{\mu }_{p}{\bar{u}}_{p}$$

5 $$\frac{d{\bar{a}}_{s}}{dt}=-{\bar{\Gamma }}_{s}{\bar{a}}_{s}-j2\eta {\bar{a}}_{p}^{\dagger }{\bar{a}}_{p}{\bar{a}}_{s}-j\zeta {\bar{a}}_{I}^{\dagger }{\bar{a}}_{p}{\bar{a}}_{p}-j{\gamma }_{s}{\bar{b}}_{s}-j{\mu }_{s}{\bar{u}}_{s}$$

6 $$\frac{d{\bar{a}}_{I}^{\dagger }}{dt}=-{\bar{\Gamma }}_{I}{\bar{a}}_{I}^{\dagger }+j2\eta {\bar{a}}_{I}^{\dagger }{\bar{a}}_{p}^{\dagger }{\bar{a}}_{p}+j\zeta {\bar{a}}_{p}^{\dagger }{\bar{a}}_{p}^{\dagger }{\bar{a}}_{s}+j{\gamma }_{I}{\bar{b}}_{I}^{\dagger }+j{\mu }_{I}{\bar{u}}_{I}^{\dagger }$$

where, $\,{{\Gamma }}_{i}=0.5({{\Gamma }}_{c,i}+{{\Gamma }}_{l,i})$, ${\gamma }_{i}=\sqrt{{\Gamma }_{l,i}}$ and ${\mu }_{i}=\sqrt{{\Gamma }_{l,i}}$ (*i* = *p*, *s*, *I*), respectively. Equations (4–6) intrinsically assume both the energy and momentum conservation principles. However, we can arrange Eqs (5 and 6) in matrix form as:

7 $$\frac{d}{dt}[\begin{array}{c}{\tilde{a}}_{s}(t)\\ {\tilde{a}}_{I}^{\dagger }(t)\end{array}]={\boldsymbol{M}}[\begin{array}{c}{\tilde{a}}_{s}(t)\\ {\tilde{a}}_{I}^{\dagger }(t)\end{array}]+{\boldsymbol{D}}(t)$$

where the variable change ${\tilde{a}}_{s}(t)={\bar{a}}_{s}{e}^{j\Delta {\omega }_{p}t}$ and ${\tilde{a}}_{I}^{\dagger }(t)={\bar{a}}_{I}^{\dagger }{e}^{-j\Delta {\omega }_{p}t}$ has been adopted, and the 2 × 2 ***M*** matrix and the driving column vector ***D***(*t*) are defined in refs 29 and 30.

In general, the pump mode can be treated as a classical field and then it can be well approximated by its expectation value: ${\bar{a}}_{p}(t)\to {\bar{a}}_{p}(t)={\bar{\alpha }}_{p}(t)$, and Eqs (4) and (7) can be solved numerically (see refs 29 and 30 for details). Then, the field operator for the photons traveling in the bus waveguide after the waveguide-resonator interaction can be found using Eq. (8):

8 $${\bar{f}}_{i}={\bar{b}}_{i}-j{\gamma }_{i}{\bar{a}}_{i}$$

At this stage, according to the definitions given in refs 16 and 19, we can calculate the first (self-correlation) and second-order (cross-correlation) temporal correlation functions, ${g}_{i}^{(1)}(\tau )$ (*i* = *s*, *I*), and ${g}_{s/I}^{(2)}(\tau )$, respectively. The time *τ* represents the temporal delay between the idler and signal photon detection.

## Results

The aim of this work is to theoretically demonstrate the feasibility of biphoton generation in an Al_x_Ga_1−x_N microring resonator operating in the UV-visible range. This is accomplished using two main steps: First, we investigate the dispersion and coupling engineering to obtain efficient phase matching and mode coupling for the SFWM process. These obtained results, which represent innovations in dispersion engineering, phase matching, and coupling in the less explored UV-visible range, are used in the second step to evaluate the fundamental statistics of quantum light. In particular, we adjust the pump wavelength such that the signal wavelength appears near the transition of one of the ions used for ion-trap.

Generation of such a correlated photon pair allows heralded single photon absorption by an ion, enabling quantum state transfer from the single photon to the ion while monitoring this quantum state transfer by the heralded photon^25^. For our analysis we select a signal photon wavelength of 369.5 nm that corresponds to the transition wavelength of ^171^Yb^+^ a promising ion for ion-trap qubit systems^21, 22^. We also constraint the pump wavelength to fall in the blue range where compact coherent diode lasers exist. In this sense, the interaction of the signal with the ion can result in a “signature” that can be detected through the detection of the idler photon at a longer wavelength in the far blue or green range. Hereafter the AlGaN alloy composition x = 0.65 is assumed in our analysis, and the Sellmeier’s index equations for Al_x_Ga_1−x_N and AlN are used to take into account the index dispersion^26^.

The conservation of energy and momentum requires that the relationships 2*ω*_*p*_ = *ω*_*s*_ + *ω*_*I*_, and 2*m*_*p*_ = *m*_*s*_ + *m*_*I*_, are fulfilled, respectively. Therefore, we need to engineer the ring resonator cross section in order to realize the phase-matching condition (which includes both momentum and energy conservation conditions), and then to optimize the SFWM process. As a result, the condition ${\rm{\Delta }}k={k}_{s}+{k}_{I}-2{k}_{p}=0$ must be satisfied, where *k*_*i*_ (*i* = *p*, *s*, *I*) is the propagation constant of the *i-th* mode involved in the SFWM process. In this context, the dispersion of the optical modes (i.e., their spectral spacing) is the most important aspect to be considered if spontaneous FWM will occur and produce entangled photon pairs.

**Figure 2:**
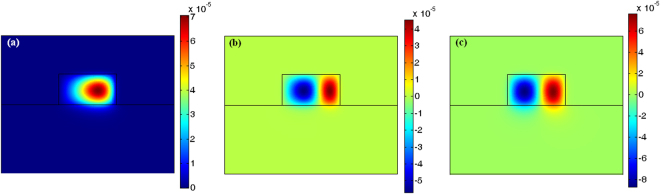
Cross section of the electric field mode profile distribution of an Al_0.65_Ga_0.35_N microring with a radius of 100 microns, and with a width and height of 1500 nm and 800 nm, rspectively, on an AlN substrate: (**a**) Quasi TE_00_ mode at *λ*_*p*_ = 445.01 nm; (**b**) Quasi TE_10_ mode at *λ*_*s*_ = 369.5 nm; (**c**) Quasi TE_10_ mode at *λ*_*I*_ = 559.31 nm.

A necessary requirement is to induce anomalous group velocity dispersion (GVD) at the pump wavelength. Under this condition, the material dispersion contribution becomes negative allowing the waveguide dispersion to be compensated, and the phase matching condition to be achieved^31^. However, this approach is precluded in the visible and the ultraviolet ranges because the GVD is strongly in the normal regime. To overcome this challenge, we use a higher-order mode for the signal and idler wavelengths and show that they can fulfil the phase matching condition, Δ*k* = 0. As a result, we choose to use the fundamental TE (TE_00_) mode in the blue range for the pump mode. Accordingly in Fig. 2(a) the TE_00_ electric field distribution in the plane of the conformal transformation^27^ is shown.

The simulation has been performed assuming *H* = 800 nm, *W*_*R*_ = 1500 nm, *R* = 100 µm, and λ_*p*_ = 445.01 nm. However, to maximize the mode overlap *ζ* we need to choose proper high-order modes. As a result, both signal and idler modes must hold the same symmetric or anti-symmetric electric field distribution. Figure 2(b) and (c) show the anti-symmetric TE_10_ modes at the signal and idler wavelengths (λ_*s*_ = 369.5 nm, λ_*I*_ = 559.310 nm), respectively.

**Figure 3:**
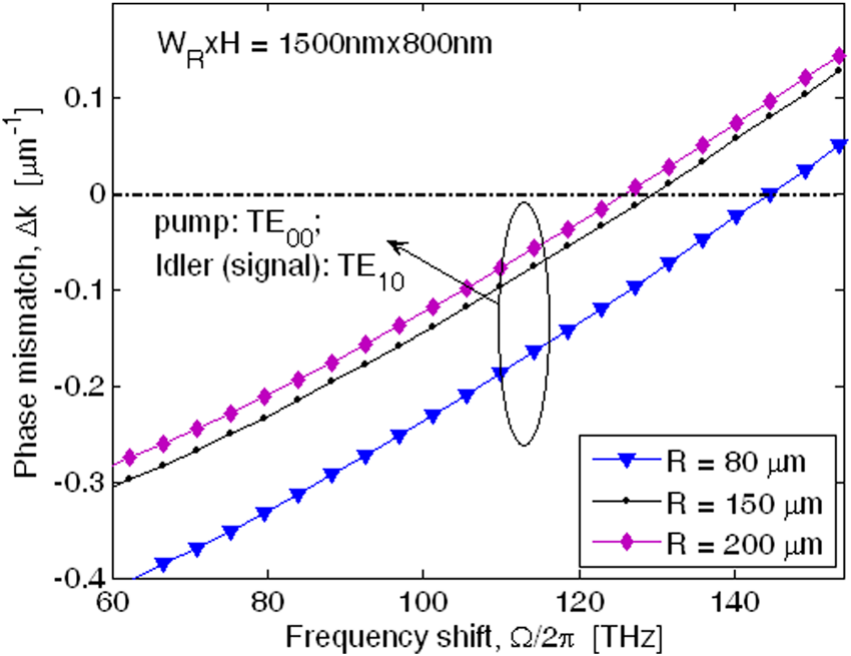
Phase mismatching parameters as a function of the signal-pump frequency shift for different values of ring radius, assuming *W*_*R*_ × *H* = 1500 nm × 800 nm, and λ_*s*_ = 369.5 nm.

Figure 3 shows the phase mismatching parameter, Δ*k*, as a function of frequency shift between the pump and signal waves, Ω/2π, (Ω = *ω*_*s*_ − *ω*_*p*_ = *ω*_*p*_ − *ω*_*I*_) for different values of the ring radius, R. Numerical results have been obtained by assuming *H* = 800 nm, *W*_*R*_ = 1500 nm, with the pump wave aligned as the TE_00_ mode and the generated signal and idler modes polarized as TE_10_. The plot of Fig. 3 reveals that the phase matching condition Δ*k* = 0 can be obtained for larger frequency shift with decreasing ring radius. In particular, we find that Ω/2π changes from 144.516 to 125.957 THz, with R ranging from 80 to 200 µm. In Table 1 the wavelengths satisfying the phase matching condition and the energy conservation relevant to the curves of Fig. 3 are summarized.

**Table 1:** Phase matching condition and energy conservation.

Ring radius R [µm]	Wavelength			Effective Refractive index		
	*λ*_*s*_ [nm]	*λ*_*p*_ [nm]	*λ*_*I*_ [nm]	${n}_{R,eff}^{(s)}$	${n}_{R,eff}^{(p)}$	${n}_{R,eff}^{(I)}$
80	369.5	449.511	573.753	2.2519	2.1975	2.1129
150	369.5	439.641	542.652	2.2623	2.2113	2.1364
200	369.5	437.349	535.720	2.2653	2.2152	2.1425

**Figure 4:**
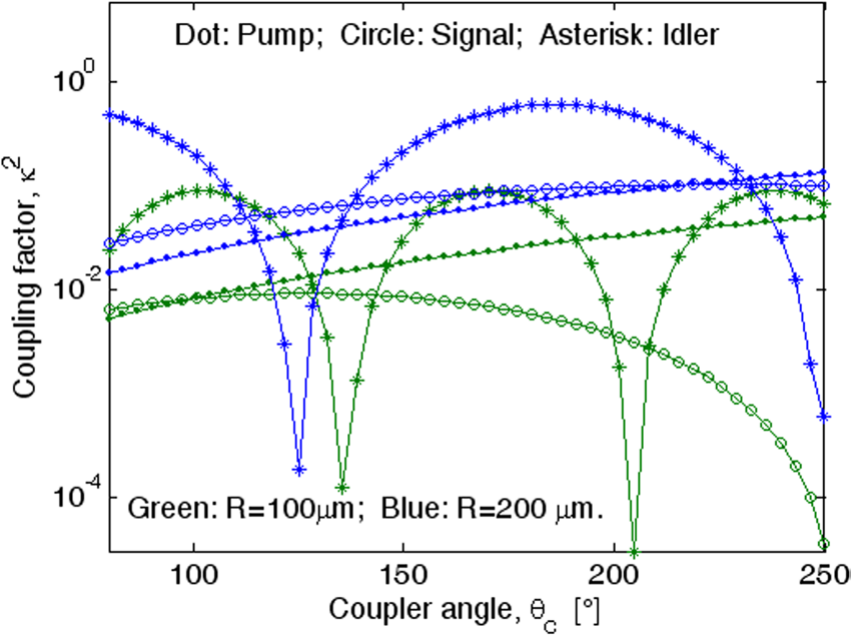
Coupling factors versus the coupler angle relevant to pump, signal and idler waves; *W*_*R*_ × *H* = 1500 nm × 800 nm, *G* = 100 nm; λ_*s*_ = 369.5 nm, and different ring radius.

Figure 4 shows the coupling factors for different ring radii, assuming *H* = 800 nm, *W*_*R*_ = 1500 nm, and *G* = 100 nm. The simulations have been performed by considering the pump wave aligned as the TE_00_ mode and the generated signal and idler modes polarized as TE_10_. Although the ring resonator is not phase matched with the bus waveguide at *λ*_*I*_ (*λ*_*s*_), the plot reveals that the idler coupling factor presents a periodic shape reaching values significantly larger than those obtained at *λ*_*p*_, where the coupler design has been optimized. This is primarily due to the fact that at *λ*_*I*_ > *λ*_*p*_ the larger interaction between the optical field tails and the coupler perturbed region partially compensates the detrimental effect induced by the phase difference between the two arms of the optical coupler.

In Table 2 we list some specific values obtained from the curves of Fig. 4. In particular, for each value of the ring radius, we have reported the critical coupling factors (${\kappa }_{i,cr}^{2}$, *i* = *p*, *s*, *I*) related to an unloaded cavity quality factor *Q*_*l*_ = 6 × 10^5^, the value of the outer waveguide width satisfying the synchronism at *λ*_*p*_ (see Methods Section), and the critical coupler angles ${\theta }_{c}^{i,cr}$ realizing the condition ${\kappa }_{i}^{2}={\kappa }_{i,cr}^{2}$. It is worth noting that in the case under investigation, *H* = 800 nm, *W*_*R*_ = 1500 nm and *G* = 100 nm, for ring resonators with *R* = 100, and 150 µm, the signal coupling factors move into the under coupling regime, ${\kappa }_{s}^{2} < {\kappa }_{s,cr}^{2}$ and for this reason the ${\theta }_{c}^{s,cr}$ values are not indicated in Table 2.

**Table 2:** Coupler parameters.

Ring radius R [µm]	Critical coupling factor			Bus waveguide width *W* [nm]	Critical coupler angle		
	${\kappa }_{s,cr}^{2}$	${\kappa }_{p,cr}^{2}$	${\kappa }_{I,cr}^{2}$		${\theta }_{c}^{s,cr}$[°]	${\theta }_{c}^{p,cr}$[°]	${\theta }_{c}^{I,cr}$[°]
100	0.0480	0.0368	0.0278	1369.0	—	~214°	~123°, ~149°, ~192°, ~217°
150	0.0722	0.0561	0.0433	1437.3	—	~202°	~103°, ~167°, ~193°
200	0.0964	0.0754	0.0587	1476.1	~192°	~186°	~111°, ~137°, ~236°

**Figure 5:**
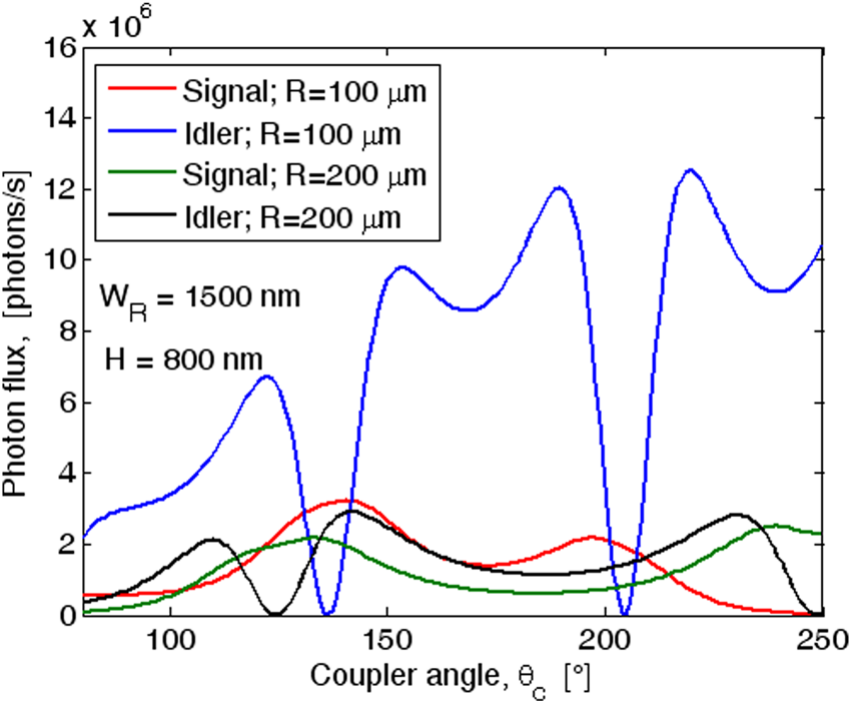
Photon flux as a function of the coupler angle, for different ring radius values, assuming *W*_*R*_ × *H* = 1500 nm × 800 nm, *G* = 100 nm, *P*_*in*_ = 25 mW, *Q*_*l*_ = 6 × 10^5^, and *λ*_*s*_ = 369.5 nm.

The signal (idler) photon flux, ${g}_{s}^{(1)}(0)$ (${g}_{I}^{(1)}(0)$), is shown in Fig. 5 as a function of the coupler angle and for different values of the ring radius. The simulations have been performed assuming *H* = 800 nm, *W*_*R*_ = 1500 nm, *G* = 100 nm, *P*_*in*_ = 25 mW, *n*_2_ = 3 × 10^−19^ m^2^/W, with the pump resonance frequency mismatch Δ*ω*_*p*_ = 0, and *Q*_*l*_ = 6 × 10^5^. While such a technologically viable Q value will generate bi-photons with a linewidth much broader than the atomic transition linewidths, resulting in less efficient atom-photon interaction, the intent of this study is to show what the pair generation rate is with such level of Q. Increasing the Q value will increase the pair generation rate and will enhance the efficiency of atom-photon interaction, but that is not the subject of this study.

The plots in Fig. 5 presents several peaks and deeps, as mainly induced by the different coupling regime occurring at the idler wavelength. Indeed, although the ring resonator is not synchronous with the bus waveguide at *λ*_*I*_ (*λ*_*s*_), the idler coupling factor presents a periodic shape reaching values significantly larger than those obtained at *λ*_*p*_, where the coupler design has been optimized (see the Methods Section). This is primarily due to the fact that at *λ*_*I*_ > *λ*_*p*_ the larger interaction between the optical field tails and the coupler perturbed region partially compensates the detrimental effect induced by the phase difference between the two arms of the optical coupler. As a result at *λ*_*I*_, the coupler works alternatively in the under-coupling, critical and over-coupling regimes, as evidenced by Fig. (4) and Table 2.

**Figure 6:**
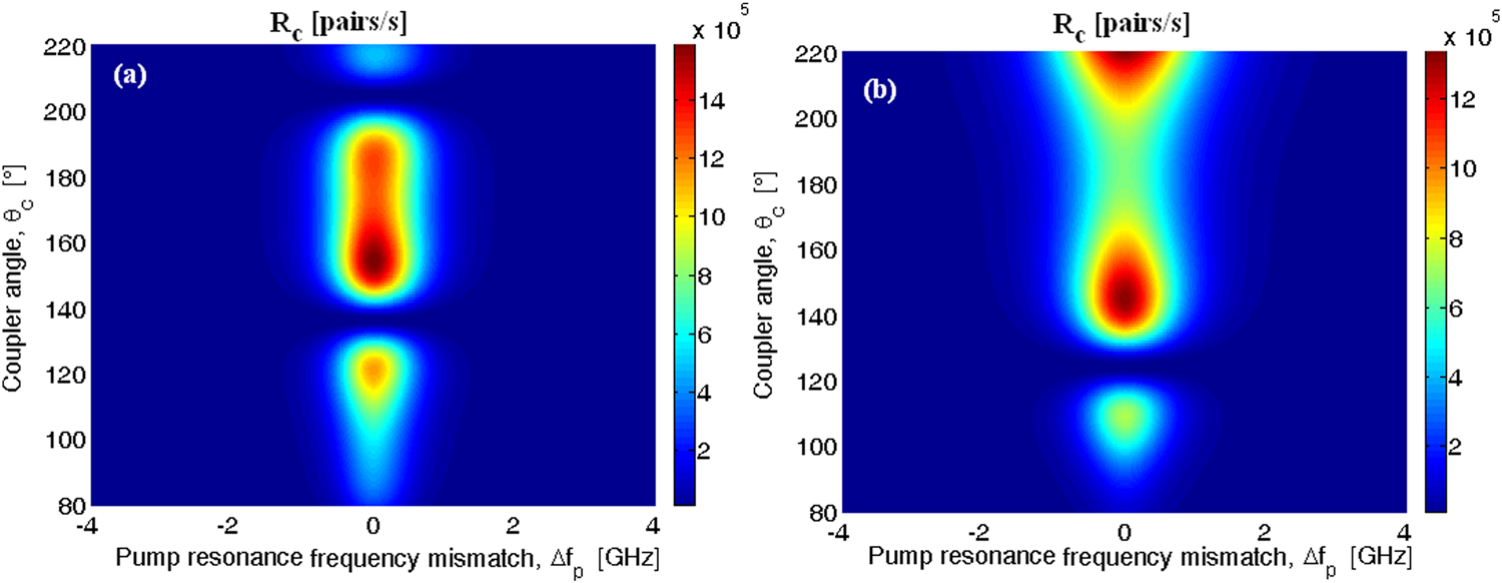
Biphoton flux as a function of the pump resonance mismatch frequency and coupler angle: (a) Signal (idler) TE_10_ mode, *W*_*R*_ x *H* = 1500 nm × 800 nm, *G* = 100 nm, and *R* = 100 µm; (**b**) Signal (idler) TE_10_ mode, *W*_*R*_ × *H* = 1500 nm × 800 nm, *G* = 100 nm, and *R* = 200 µm.

According to ref. 19, we evaluate the average photon-pair flux, *R*_*c*_, by integrating the function ${|{R}_{s/I}(\tau )|}^{2}={g}_{s/I}^{(2)}(\tau )-{g}_{s}^{(1)}(0)\cdot {g}_{I}^{(1)}(0)\,$ over the time τ. Under this definition, in Fig. 6 the average biphoton flux (*R*_*c*_) is sketched as a function of the pump resonance frequency mismatch (Δ*f*_*p*_ = Δ*ω*_*p*_/2*π*) and the coupler angle, setting the input pump power *P*_*in*_ = 25 mW, and the Kerr coefficient *n*_2_ = 3 × 10^−19^ m^2^/W. In the simulations, we have assumed the following parameters: (a) *H* = 800 nm, *W*_*R*_ = 1500 nm, *G* = 100 nm, and *R* = 100 µm; (b) *H* = 800 nm, *W*_*R*_ = 1500 nm, *G* = 100 nm, and *R* = 200 µm. Moreover, in order to realize a comparison among the architectures considered, we have assumed for each case a fixed unloaded quality factor.

It is interesting to note that the plot of Fig. 6 allows us to find the optimum coupler angle required to maximize the average biphoton flux for a given input pump power. The presence of multiple peaks is mainly due to the periodic shape induced in the idler coupling factor (see Fig. 4). In the cases of Fig. 6(a) and (b), the maximum produced biphoton flux (*R*_*c*,*max*_) assumes values of 1.59 × 10^6^, and 1.31 × 10^6^ pairs/s for *θ*_*c*_ equal to 154° and 144°, respectively. Moreover, our simulations indicate that the cross correlation function, ${g}_{s/I}^{(2)}(\tau )/({g}_{s}^{(1)}(0)\cdot {g}_{I}^{(1)}(0))$, between signal and idler photons increases with a decrease in the pair flux, reaching a peak value for coupler angle values different from those inducing *R*_*c*,*max*_. In particular, we record a peak value for ${g}_{s/I}^{(2)}(\tau )/({g}_{s}^{(1)}(0)\cdot {g}_{I}^{(1)}(0))$ of 1680 and 2.37 × 10^4^, in correspondence with *θ*_*c*_ equal to 97° (*R* = 100 µm) and 80° (*R* = 200 µm), respectively, for *P*_*in*_ = 25 mW and Δ*f*_*p*_ = 0, where the relevant biphoton flux assumes values of 5.368 × 10^5^ and 8.784 × 10^4^ pairs/s, respectively. These values are comparable to those reported in ref. 16 in which a silicon microdisk resonator has been pumped at telecom wavelength. However, if compared with the silicon technology, our device based on the Al_x_Ga_1−x_N platform presents a reduced cavity enhancement factor and a reduced nonlinear strength coefficient, as a result of: (1) using higher order modes to induce the phase matching, (2) the reduced index contrast, and (3) the smaller Kerr coefficient, and (4) larger mode volume due to a larger resonator radius considered in our work. Consequently, an increase of input pump power is needed to induce a significant SFWM effect. In any case, the devices proposed in this work show the non-trivial features needed to induce entanglement photons in the visible wavelength range where the silicon cannot operate.

**Figure 7:**
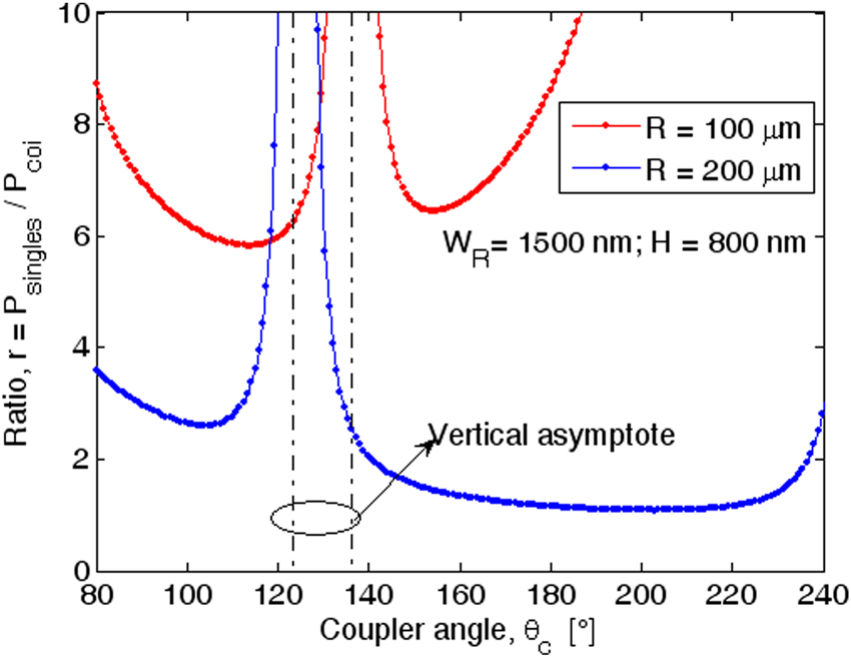
Ratio *r* as a function of the coupling angle for different ring radii: Signal (idler) TE_10_ mode, *W*_*R*_ × *H* = 1500 nm × 800 nm, *G* = 100 nm.

In Fig. (7) we show the ratio *r* = *P*_*singles*_/*P*_*coi*_, between the total singles probability (*P*_*singles*_) and the coincidences rate (*P*_*coi*_), as a function of the coupler angle for different values of the ring resonator radius, assuming *P*_*in*_ = 25 mW, *H* = 800 nm, *W*_*R*_ = 1500 nm, and *G* = 100 nm. According to the formulas proposed in refs 29 and 32, the plot evidences vertical asymptotes relevant to the condition ${\kappa }_{i}^{2}=0\,$(*i* = *s*, *I*), that, in our case, occurs only for idler photons. As outlined in ref. 29, *r* =  2 is considered the theoretical value obtained in the condition for which both signal and idler are at the critical coupling regime. Moreover, a reduction of *r* can be obtained in the over-coupling regime The plot of Fig. 7 indicates that for *R* = 100 µm, the condition *r* > 2 is met for all values of the coupler angle, due to the fact that the signal moves always in the under-coupling regime (see Table 2). In addition, two minimum values of *r* are obtained close to the idler critical coupling angle. Quite different are the features in the case of *R* = 200 µm, where *r* < 2 can be obtained for a coupling angle in which both signal and idler are in the over-coupling regime.

At this stage, some comments about the signal (idler) resonance frequency mismatch are worth making. It is legitimate to assume that the signal (idler) spectrum can be well approximated by the product of two Lorentzian functions, characterized by a parameter *ρ*, which can be either purely real, purely imaginary, or exactly zero, depending on the input pump power, the Kerr coefficient and the pump resonance frequency mismatch. Generally speaking, the parameter *ρ* depends on the numerical solution of Eq. (7), however a robust estimation is given in ref. 30. In this context, the two Lorentzian functions show identical spectral widths *δω* centered at ${\rm{\Delta }}{\omega }_{s}({\rm{\Delta }}{\omega }_{I})={\rm{\Delta }}{\omega }_{p}\pm \rho$, where *ρ* is imaginary. In addition, to preserve the energy conservation if, for example, the signal photons are generated at the resonance mismatch ${\rm{\Delta }}{\omega }_{s}={\rm{\Delta }}{\omega }_{p}+\rho$, then in that case the generated idler photons will suffer from ${\rm{\Delta }}{\omega }_{I}={\rm{\Delta }}{\omega }_{p}-\rho$.

**Figure 8:**
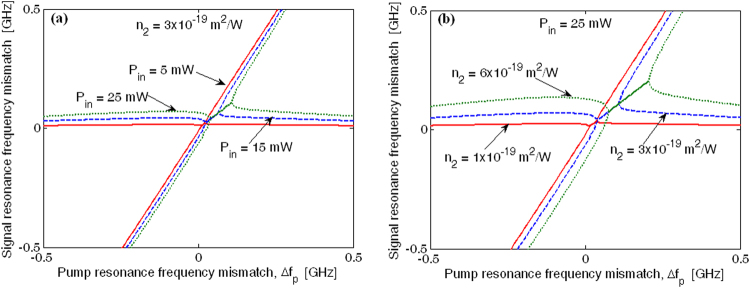
Signal resonance frequency mismatch as a function of the pump resonance frequency mismatch; (**a**) different values of the input pump power with *Q*_*l*_ = 6 × 10^5^, and *n*_2_ = 3 × 10^−19^ m^2^/W; (**b**) different values of the Kerr coefficient with *Q*_*l*_ = 6 × 10^5^, and *P*_*in*_ = 25 mW.

Conversely as *ρ* becomes real, the lineshape takes the form of the product of two Lorentzian functions, both centered at ${\rm{\Delta }}{\omega }_{s}({\rm{\Delta }}{\omega }_{I})={\rm{\Delta }}{\omega }_{p}$ but having spectral widths *δω* = Γ_*m*_ ± *ρ*. In this instance, Fig. 8(a) and (b) plot the signal resonance frequency mismatch as a function of the pump resonance frequency mismatch, for different values of the input pump power and Kerr coefficient, respectively. The simulations have been performed considering a device having *H* = 800 nm, *W*_*R*_ = 1500 nm, *W* = 1369 nm, *G* = 100 nm, *R* = 100 µm, and *θ*_*c*_ = 110°. All plots clearly show the frequency splitting effect and the region (rectilinear shape) where the parameter *ρ* is real. Moreover, we can observe that the no-splitting region width increases with the input pump power and the Kerr coefficient, shifting towards larger positive values of Δ*f*_*p*_.

## Conclusions

We have proposed and analysed the SFWM generation of entangled bi-photon quantum light sources in the UV-vis range using AlGaN microring resonators. These bi-photons can be used for scalable ion-trap qubits that have their transition wavelengths in the UV-vis range. In this work, we designed pump/signal/idler photons to be broadly spaced in the wavelength spectrum such that the pump wavelength falls in the blue wavelength regime where compact coherent III-Nitride laser sources exist, where the signal wavelength coincides with the transition wavelength of ytterbium ion (^171^Yb^+^, 369.5 nm), and where the idler wavelength resides in the far blue or the green spectrum. Though the material dispersion of most optical materials, including AlGaN, are strongly normal in the UV-vis range, we have shown that by using higher order modes of an AlGaN microring resonator we can overcome the normal dispersion enabling the phase matching condition for efficient generation of bi-photons. We implemented a mathematical modeling to investigate the correlation and the flux rate of the entangled biphotons in AlGaN high-Q microring resonators. Numerical simulations have revealed cross correlation values of 2.37 × 10^4^ at a biphoton flux of 8.748 × 10^4^, for a ring radius *R* = 200 µm, a resonator cross section *W*_*R*_ × *H* = 1500 nm × 800 nm, and *P*_*in*_ = 25 mW. The signal and idler photons are generated at wavelengths of 369.5 and 535.72 nm, respectively. Integration of such a AlGaN microring resonator, which is a III-Nitride material, with III-Nitride blue lasers on-chip, opens up a new avenue for scalable heralded single photon absorption by single ions used in quantum information technology.

## Methods

As outlined in the Theory Section, the entanglement features of the microring system are strongly influenced by the photon rate coefficients. In this sense, we have focused our investigation on estimating the coupling factors for pump, signal and idler waves in order to realize a good trade-off between the average biphoton flux and the cross-correlation functions. As discussed earlier, we use pulley coupling between the waveguide and the resonator. We model the pulley coupler using the conformal transformation^27^, and evaluating the coupling coefficient as:

9 $${K}_{c}=\omega \langle {e}_{R}|{\rm{\Delta }}\varepsilon |{e}_{B}\rangle /(4\sqrt{\langle {e}_{R}|{e}_{R}\rangle \langle {e}_{B}|{e}_{B}\rangle })$$

where Δ*ε* is the dielectric perturbation, while 〈*e*_*R*_| and 〈*e*_*B*_| denote the modes in the ring and bus waveguide, respectively.

Note that the physical path lengths in the concentric coupled sections of the ring resonator and the external bus waveguide are different. Therefore to maintain phase matching between the waves traveling into the resonator and the external bus, the two waveguides must be different in such a way that *n*_*R*,*eff*_ *R* = *n*_*B*,*eff*_(*R* + *W* + *G*), where *n*_*R*(*B*),*eff*_ is the effective refractive index in the ring resonator (external bus), *R*, *G* and *W* are the ring radius, the coupling gap and the outer waveguide width, respectively (see Fig. 1(a)). This can be achieved by varying the width of the outer waveguide under the condition *W*_*R*_ > *W*.

The numerical calculations for waveguide-resonator coupling run as follows: first, at the pump wavelength and for given values of *W*_*R*_, *G*, and *R* we use FEM simulations and conformal transformation to determine the ring resonator refractive index and the outer waveguide width *W* satisfying the relationship *n*_*R*,*eff*_ *R* = *n*_*B*,*eff*_(*R* + *W* + *G*). Then, the coupling coefficient *K*_*c*_ is calculated by means of Eq. (9), evaluating the electric field overlap integrals from FEM simulations for each wave involved in the SFWM process. Finally, according to refs 27 and 33, the coupling factors relevant to the three waves involved in the SFWM process are estimated as a function of the coupling angle, θ_c_ (see Fig. 1(a)). Moreover, the phase mismatching between the waves travelling into the ring resonator and the bus waveguide occurring at the signal and idler wavelengths is also considered.
